# Recommended Apps for 2015

**Published:** 2015-07-01

**Authors:** Wendy H. Vogel

**Affiliations:** Ms. Vogel is a nurse practitioner at Wellmont Cancer Institute in Kingsport, Tennessee.

There is a whole world of smartphone apps out there. It’s impossible to weed through all of them. Some of the best apps I have were recommended by a friend or colleague or even an airplane seatmate! So I asked some of our *Journal of the Advanced Practitioner in Oncology* (JADPRO) readers about their favorite smartphone apps, and we came up with some really useful ones. All of these apps are available on Apple, Google, and Windows platforms unless otherwise specified.

**About Herbs** • https://www.mskcc.org/apps *(free)* From Memorial Sloan Kettering Cancer Center’s integrative medicine service, this app is only available as an Apple app, but a Web app version for all other mobile devices is also available. This app provides comprehensive, objective information about herbs, botanicals, supplements, complementary therapies, and more. There are two different versions of each monograph: one written for health-care professionals and one written for consumers. You can search offline if the app is downloaded. This is one of my favorite apps, and I use it almost daily. I also direct patients to this app as well.

**Ask The Nutritionist: Recipes for Fighting Cancer** • dana-farber.org/nutrition-app.aspx *(free)*. From this Dana-Farber Cancer Institute app, you can obtain healthy diets/recipes and nutrition tips for managing cancer side effects and for living healthy during treatment and afterward. Users can ask a nutritionist questions or search the database of frequently asked questions. The database can be searched by food types, meal types, special diets, or symptom management. The app is meant to be used by practitioners and patients/survivors.

**CTCAE v4.0** *For Apple devices ($1.99)* The National Cancer Institute Common Terminology Criteria for Adverse Events (CTCAE) is easily accessed with this mobile app. It allows for bedside/chairside grading of toxicities. It is easy to navigate by body systems. You can bookmark selected adverse events/categories for quick access and search for events across names, definitions, and grades. I found this app much easier to search than the paper or online copy. There is a similar app on Google Play called CTCAE 4.03 (free). A Windows version—CTCAE: Common Terminology Criteria for Adverse Events 4.0—is also available for $2.99. If you have used the Google or Windows version and liked it, please let us know!

**Electronic Preventive Services Selector (ePSS)** • epss.ahrq.gov/ePSS/ *(free)* This app is designed to help primary care clinicians identify clinical preventive services that are appropriate for their patients. You can search and browse the US Preventive Services Task Force (USPSTF) recommendations on the Web or on your mobile device.

**Medscape** • www.medscape.com/public/mobileapp *(free)* This app provides the latest medical news including journal articles, US Food and Drug Administration announcements, and conference news. Pediatric and adult drug information is easily available and searchable. There are a drug interaction tool and medical calculators. Formulary information can also be accessed. Kelley Mayden, MSN, FNP, AOCNP®, says some of the things she likes about this app is that CME is available via the app and the anatomy section that she uses to educate both patients and staff. She also uses the pill identifier to assist patients when they bring in their medication box but aren’t sure what is in it!

**PathLead** • www.leicabiosystems.com/landing-pages/ppc/pathlead-asr-mobile-app/ *(free)* This is an immunohistochemistry educational resource for both the novice and experienced practitioner. Kathy Sharp, MSN, FNP, AOCNP®, CCD, is very pleased with the amount of information this app contains. It lists useful organ system panels. In addition, the antibody citation index is available offline. There are webinars on histology, immunohistochemistry, compliance, and more. Short lectures can be accessed on YouTube or downloaded.

**DuoLingo** • duolingo.com *(free)* Always wanted to learn a foreign language? Clear your head during breaks by practicing some French for your (real or imagined!) Parisian holiday. You can choose your path with sessions of 5, 10, or 15 minutes per day. You can start with the basics, or if you already have some grasp of the language, you may take a placement test to see where you should start. Languages include Spanish, French, German, Italian, Portuguese, Dutch, Swedish, Danish, Irish, and Turkish. There is also an immersion section in which you can practice reading and translating articles into your selected language. DuoLingo won the 2013 iPhone App of the Year and 2014 Google’s Best of the Best awards.

**Evernote** • evernote.com *(free; upgrade for $5 to $10 per month)* Here is a professional workspace for writing, collecting information, and presenting ideas. You can clip Web articles, capture handwritten notes, and utilize photos. It is easily searchable; transforms notes into slides; and syncs across smartphone, tablet, and computer. I use Evernote for remembering chemotherapy regimens, diagnostic workups, and more at work. At home, I use it for recipes, passwords, personal notes, to-do lists, and more. Notes can be sent to other users by e-mail.

**SimplyRain** • simplynoise.com *($0.99)* This is a refreshing way to relax, listening to rainfall. You can control the intensity, including thunder and wind, and use a sleep timer. The maker, SimplyNoise, has several other apps, including a babbling brook and ocean waves. For Google Play, the app is called SimplyNoise (also $0.99) and has various white-noise offerings.

**Coffitivity** • coffitivity.com *(free)* This app provides you with the energy and creativity of working in a cafe, without having to leave your home or office! Coffitivity recreates the ambient noises of a cafe. Several different audio tracks are available, and you can open your favorite music from this app. It can also be used without the Internet access.

**Pomodoro** • pomodorotechnique.com *(free; $1.99, $2.99)* This app helps you boost your productivity using the Pomodoro Technique®, one of the most effective time-management methods out there. The principle behind it is to have you focus for a short, productive period of time, and it reminds you to take a short break and then return to focusing on your work again. There are several similar apps available for different devices. The free versions is identical to the paid version, but it has ads.

**Phocus** • phocusapp.com *($1.99)* This is another time-management app to increase your productivity. You can set your pace and track your performance. It is only available as an Apple app.

